# Health-related quality of life in children born preterm at school age: the mediating role of social support and maternal stress

**DOI:** 10.3389/fpsyg.2024.1463804

**Published:** 2024-12-02

**Authors:** Melissa Liher Martínez-Shaw, Kari Anne I. Evensen, Sandra Melero, Yolanda Sánchez-Sandoval

**Affiliations:** ^1^Department of Psychology, Faculty of Education Sciences, University of Cadiz, Cádiz, Spain; ^2^Instituto de Investigación e Innovación Biomédica de Cádiz (INiBICA), Cádiz, Spain; ^3^Department of Clinical and Molecular Medicine, Norwegian University of Science and Technology, Trondheim, Norway; ^4^Children’s Clinic, St. Olavs Hospital, Trondheim University Hospital, Trondheim, Norway; ^5^Department of Rehabilitation Science and Health Technology, Oslo Metropolitan University, Oslo, Norway

**Keywords:** preterm children, health-related quality of life, socio-family risk index, social support, maternal stress, mediation analysis

## Abstract

Research on health-related quality of life (HRQoL) of school-aged children born preterm (< 37 weeks of gestational age) is scarce and there are few studies examining the relationship with medical and family factors. The aims were to analyze HRQoL in a sample of 8-year-old children born preterm with very low birth weight (VLBW), to test a proposed theoretical model that examines the relationship with medical and socio-family factors, and to explore the mediation effects of maternal factors between perinatal variables, demographic characteristics and HRQoL. A total of 147 VLBW children and 116 mothers were assessed. The measures included for assessment were self-and parent-reported HRQoL, functional social support, maternal stress, socio-family risk index and neonatal medical risk index. Mediation analysis was applied to investigate mediation effects of the maternal factors. Mean self-and parent-reported KIDSCREEN scores were 55.1 (*SD* 10.1) and 58.2 (*SD* 9.1), respectively, indicating better HRQoL than the normed sample with a mean of 50 (*p* < 0.001). The total effect of the initial theoretical model was not significant, thus another partial model was validated. Socio-family risk index significantly influenced HRQoL (direct effect), and this relationship was mediated by functional social support and maternal stress (indirect effects). School-aged VLBW children and their parents reported better HRQoL than the mean reference value on KIDSCREEN-10 and -27. Maternal stress and social support had a mediating effect on the children’s HRQoL. These results could be used to tailor interventions in these families.

## 1 Introduction

In the last years, the survival rates following the birth of extremely low birth weight (ELBW; < 1,000 g) infants have increased (Arpino et al., 2010), although the risk of neonatal morbidities has also increased with decreasing gestational age and very low birth weight (Manuck et al., 2016; Mukherjee et al., 2023; Platt, 2014). Different health difficulties are more frequent in these children than in term-born children (Larroque et al., 2008; Marsh et al., 2023; Taylor and O’Shea, 2022; Chung et al., 2020). Extremely preterm infants are at risk of deficits with potential consequences in functionality regarding general health. Nevertheless, the impact of the unhealthy condition on the well-being of these children and their families has been poorly described (Vederhus et al., 2010; Shaw et al., 2023).

This effect can be studied on health-related quality of life (HRQoL), which is a dynamic, multidimensional, and subjective concept, associated with health status in physical, emotional, and social functioning (Hays and Reeve, 2008). HRQoL assessments provide valuable additional information to traditionally reported medical evaluations (Saigal and Doyle, 2008). Some HRQoL assessments following individuals born preterm or with low birth weight and their families have applied multi-dimensional health profile measures, including physical, psychological, and social well-being (Pal et al., 2020; Petrou et al., 2020).

Research on quality of life during the school years of very preterm or very low birth weight (VLBW) children is scarce. Two separate systematic reviews about HRQoL in preterm children include only one study each at school age (Vieira and Linhares, 2016; Zwicker and Harris, 2008). The first study (Saigal et al., 1994a; Saigal et al., 1994b) compared the HRQoL of extremely low birth weight children (ELBW) with that of a reference group, showing that the global long-term burden experienced by children with ELBW was higher, with the latter presenting more functional limitations. In the second study, the health score of ELBW children was similar to that of normal birth weight children, while parents reported significantly poorer health for their ELBW children (Hack et al., 2011).

In addition to the studies included in the systematic reviews, Peart et al. (2021) studied HRQoL in a sample of 8-year-old extremely preterm children compared with three cohorts of term-born children with different birth years. In this study, parent-reported HRQoL was lower for children born extremely preterm with respect to all control groups (Huhtala et al., 2016) concluded that HRQoL was not significantly different for healthy VLBW children at 8 years of age when compared to a control group.

Few studies have examined factors related to a better or worse HRQoL for these children during their school years. Studies included in Mottram and Holt (2010) found that preschool preterm children with lower GA had lower scores in HRQoL. Other studies have found no differences in HRQoL related to GA in preschool children (Schiariti et al., 2007), or in birth weight in healthy preterm VLBW children at school age (Huhtala et al., 2016) compared with full-term children.

There is little evidence of the mechanism through which the effects of neonatal morbidities are transmitted to HRQoL in preterm children. One study found that, among children born extremely preterm, perinatal, and neonatal morbidity was mostly unrelated to HRQoL (Vederhus et al., 2010). Recent studies have clarified the mediating effects of bronchopulmonary dysplasia (BPD) and severe non-respiratory morbidity between GA and HRQoL (Kim et al., 2023). More specifically, the mediation analysis revealed that BPD and severe non-respiratory morbidity contributed to significant reductions in HRQoL, with indirect effects translating into HRQoL score decrements for both at the gestational ages studied (<26, 26–27, and 28–29 weeks). The effect was more detrimental when the GA was lower. In this case, as the authors indicate, the worse HRQoL score is mainly due to the complications of preterm birth rather than the preterm birth itself.

Furthermore, studies about families of children with neurocognitive developmental disorders and disabilities (NDD) have highlighted a clear relationship between children’s quality of life, parents’ quality of life, and family environment, concluding that the importance of family well-being in the well-being of the child should be recognized (Puka et al., 2020). The similarity in parental quality of life among parents of children with different NDD indicates that quality of life is influenced by parental and family characteristics rather than by the disease or specific factors (Hatzmann et al., 2008; Puka et al., 2018). To the best of our knowledge, little is known about how family variables influence the quality of life of children born preterm at school age. Much more is known about maternal stress before or immediately after delivery (Hoffman et al., 2016). Another study highlighted the importance of social support as a protective factor for perinatal depression in the 2 years following postpartum and adverse child development (Milgrom et al., 2019). Socioeconomic level, ethnicity, family conflicts, maternal distress and maternal education are some risk factors identified for development in studies about children born prematurely (Moreira et al., 2014). There is greater knowledge of how family variables behave when children are newborns or toddlers, but not how they behave in the long-term during school age. It remains unclear how these variables act on each other as these children grow up, as well as the real impact on their quality of life, knowing their true mediating role.

Therefore, we aimed to (1) study HRQoL in a sample of children born preterm with VLBW at 8 years of age, (2) examine the relationship between medical and socio-family factors and HRQoL, and (3) explore the mediation effects of maternal factors between perinatal variables, socio-family characteristics and HRQoL.

**Figure 1:**
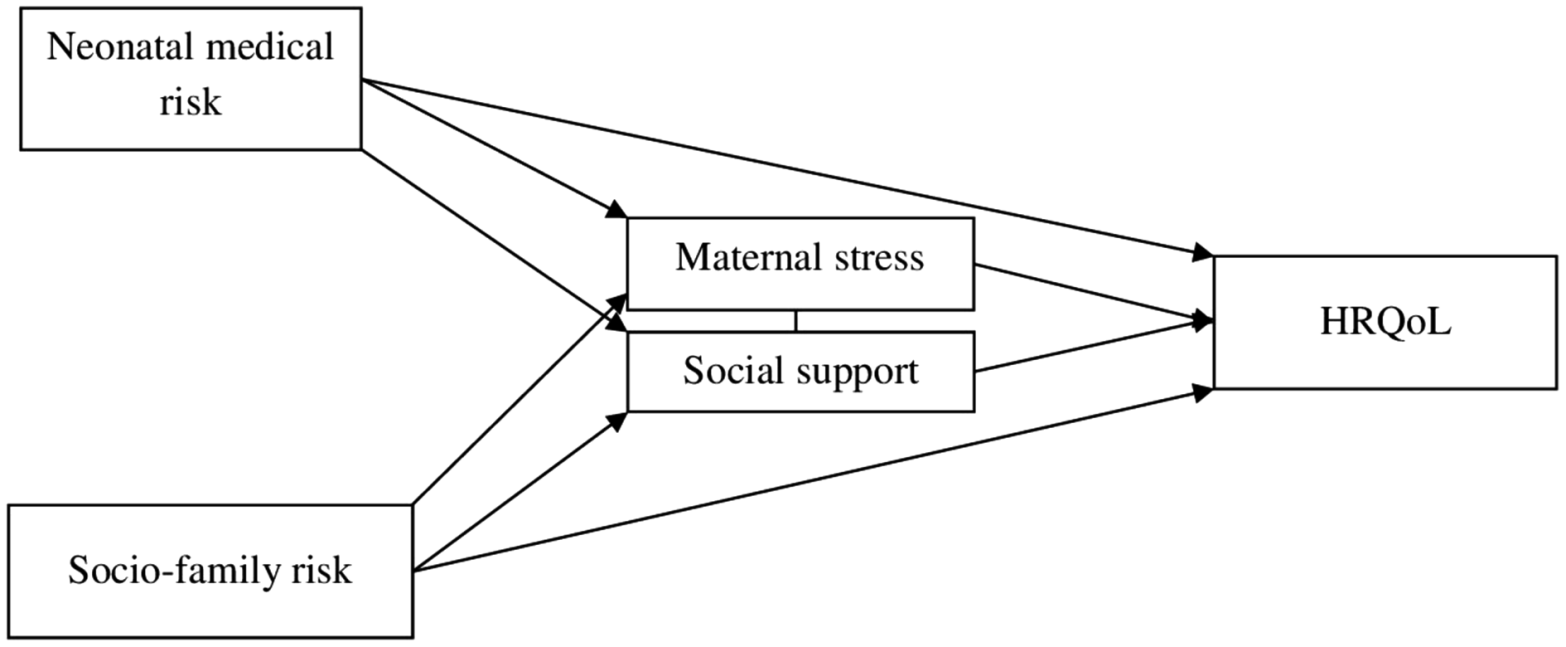
Hypothetical model of mediation analysis among neonatal medical risk, socio-family risk and HRQoL.

We proposed a theoretical model, testing a direct effect of neonatal medical and socio-family variables, as well as a mediating effect of maternal variables, on the HRQoL of preterm children (see Figure 1). This model draws on other studies (Hatzmann et al., 2009; Isa et al., 2016; Wang et al., 2022), although they did not refer specifically to preterm samples at school age. According to the model, risk factors related to children’s perinatal status and socio-family situation have direct negative effects on their HRQoL. Additionally, parental stress and perceived support would play a mediating role in the association between risk factors and quality of life. This model can be applied not only to the general quality of life, but also to its subdimensions. Within this model, partial models will be considered if the theoretically proposed model is not confirmed.

We tested the following hypotheses: (1) children in the study will show lower HRQoL than the normed mean, (2) there will be a negative association between neonatal medical and socio-family risk and the level of HRQoL, and (3) this relationship would be mediated by the level of stress and the support perceived by the mothers.

## 2 Methods

### 2.1 Participants

This prospective two-wave study was conducted from a cohort of children born preterm with very low birth weight (<1,500 g). These children were admitted to the neonatal intensive care unit (NICU) of the Puerta del Mar Hospital in Cádiz (Spain) between the years 2011 and 2014. The exclusion criterion was the presence of major congenital malformations, chromosomal abnormalities, or congenital infections.

**Figure 2:**
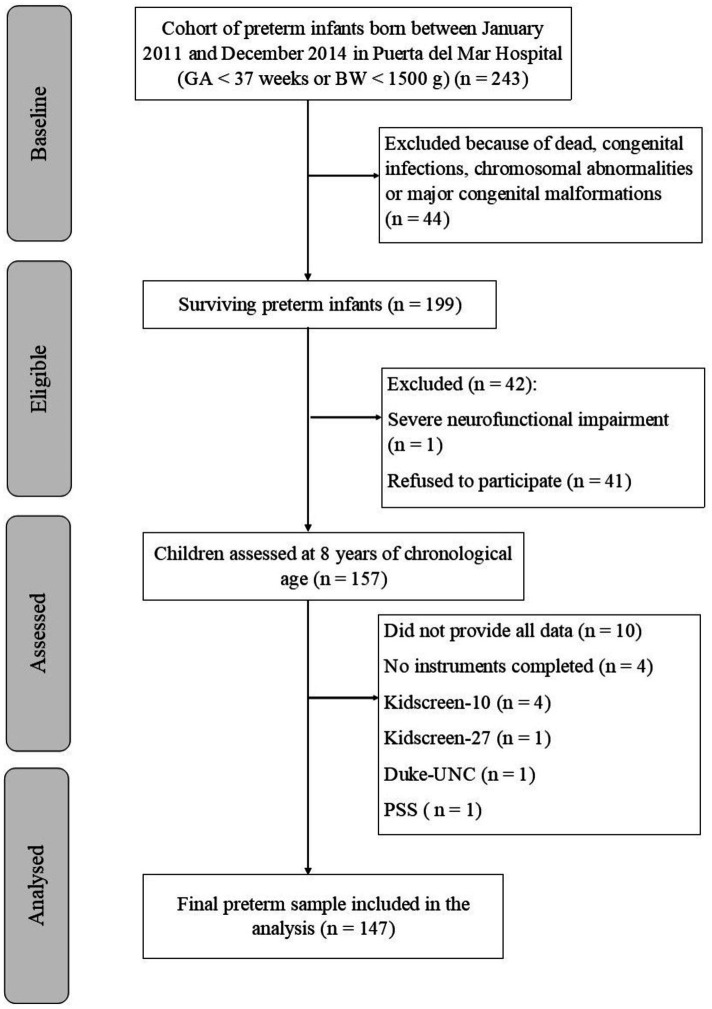
Flowchart of participants.

A total of 199 children were eligible for the assessment, of whom 147 children and 116 mothers were finally included (Figure 2). Demographic variables such as GA, birth weight (BW), sex, perinatal variables and maternal age at birth were collected at the time of birth of the child. Children’s age at present and family characteristics (family structure, education level, employment status, family income and language spoken at home) were collected at the 8-year follow-up assessment. All demographic characteristics are included in Table 1.

**Table 1:** Demographic characteristics of the sample.

Demographic variable	Total sample
Children (*n* = 147)	
Gestational age, mean weeks (*SD*)	29.81 (2.16)
Birth weight, mean grams (*SD*)	1,288.41 (362.16)
Female, *n* (%)	70 (47.6)
SGA, *n* (%)	17 (11.6)
Multiple birth, *n* (%)	68 (46.3)
IVF, *n* (%)	37 (25.2)
Cesarean sections, *n* (%)	106 (72.1)
Apgar score 1 min, mean (*SD*)^a^	6.58 (1.74)
Apgar score 5 min, mean (*SD*)^a^	8.16 (1.30)
Necrotising enterocolitis, *n* (%)	0 (0.0)
Bronchopulmonary dysplasia, *n* (%)	5 (3.4)
Patent ductus arteriosus, *n* (%)	5 (3.4)
Grade 3+ retinopathy of prematurity, *n* (%)	3 (2.0)
Severe brain injury, *n* (%)	1 (0.7)
Late-onset sepsis, *n* (%)	24 (16.3)
Neonatal medical risk index, mean [range]	0.26 [0–3]
Higher neonatal medical risk index, *n* (%)	31 (21.1)
Age at the time of assessment, mean years (*SD*)	8.66 (0.96)
Mothers (*n* = 116)	
**Family structure**	
Biparental, *n* (%)	89 (76.7)
Separated parents, joint custody, or reconstituted family, *n* (%)	11 (9.5)
Single caregiver, *n* (%)	16 (13.8)
**Education level**	
University (>12 years), *n* (%)	38 (32.8)
Semi-qualified (11–12 years), *n* (%)	40 (34.5)
Formal (<11 years), *n* (%)	38 (32.8)
**Employment status of both caregivers**	
Employment, *n* (%)	68 (58.6)
Employment of one of the caregivers, *n* (%)	44 (37.9)
Unemployment/pension, *n* (%)	4 (3.4)
**Family income**	
≥1,801 €, *n* (%)	61 (52.6)
901–1,800 €, *n* (%)	42 (36.2)
<900 €, *n* (%)	13 (11.2)
**Language spoken at home**	
Only Spanish, *n* (%)	112 (96.6)
Some Spanish, *n* (%)	2 (1.7)
No Spanish, *n* (%)	2 (1.7)
**Maternal age at child’s birth, mean years (*SD*)**	**32.98 (5.22)**
>21 years of age, *n* (%)	109 (94)
18–21 years of age, *n* (%)	5 (4.3)
<18 years of age, *n* (%)	2 (1.7)
Socio-family risk index, mean [range]	2.52 [0–9]
Age at the time of assessment, mean years (*SD*)	41.40 (5.22)

^aMean over 141 participants; SGA, small for gestational age; IVF, in vitro fertilization.^

### 2.2 Measures

The self-reported HRQoL of children was assessed using a generic measure, i.e., the KIDSCREEN-10 (Ravens-Sieberer et al., 2010), which consists of 10 items with a 5-point response scale. This instrument provides a single score of HRQoL. These values were computed into Rasch scores and transformed into t-values (*M* = 50 and *SD* = 10). The internal consistency of the scale was acceptable (*α* = 0.719).

KIDSCREEN-27 (Ravens-Sieberer et al., 2007) was also used, which assesses the proxy report HRQoL through parental response. It consists of 27 items with a 5-point Likert scale. The subscales of this instrument are physical well-being, psychological well-being, autonomy and parent relationship, social support and peers, and school environment. The reliability was excellent (*α* = 0.913), and the subdimensions had an internal consistency of 0.824, 0.768, 0.817, 0.615 and 0.751, respectively, going from questionable to good. Higher scores on KIDSCREEN measures indicate a better HRQoL.

The Duke-UNC Functional Social Support Questionnaire (Bellón et al., 1996; Broadhead et al., 1988) assessed parents’ self-perceived social support. It consists of 11 items with a 5-point Likert scale. This scale provides a global score (good internal consistency; *α* = 0.886) and two subscales (affective support and confidant support). Higher scores indicate more perceived support. The first subscale assesses perceived support as a relational catalyst (acceptable internal consistency; α = 0.765) and the second subscale assesses as a source of strength (good internal consistency; α = 0.847).

The Spanish version of the Parental Stress Scale (PSS; Berry and Jones, 1995; Spanish adaptation by Oronoz et al., 2007) comprises 12 items rated on a 5-point Likert scale. It provides a total score and two subscales (baby’s rewards and stressors), with higher scores showing a higher level of parental stress. The internal consistency was acceptable (*α* = 0.758) for the total score and questionable and acceptable (*α* = 0.600 and *α* = 0.778) for the subscales.

Socio-family risk index (SFRI) is a composite measure with six social and family variables, based on previous studies (Jiménez-Luque et al., 2023; Sánchez-Sandoval and Verdugo, 2021; Yaari et al., 2019). The variables that constitute SFRI are: family structure (0 = biparental; 1 = separated parents, joint custody, or reconstituted family; 2 = single caregiver), maternal education level (0 = university; 1 = semi-qualified; 2 = formal), employment status of both caregivers (0 = employment; 1 = employment of one of the caregivers; 2 = unemployment/pension), family income (0 = more than 1,801 €; 1 = 901–1,800 €; 2 = less than 900 €), language spoken at home (0 = only Spanish; 1 = some Spanish; 2 = no Spanish), and maternal age at birth (0 = more than 21 years of age; 1 = 18–21 years old; 2 = less than 18 years of age). Depending on the risk, values from 0 to 2 were assigned to each variable, with a total index range from 0 to 12. Data were obtained from an *ad hoc* Socioeconomic Status Questionnaire (SES).

Neonatal medical risk index (NMRI; Jiménez-Luque et al., 2023) is a cumulative index that encompasses the presence of these perinatal conditions: confirmed necrotizing enterocolitis (Bell Stage II or higher), moderate-to-severe bronchopulmonary dysplasia (oxygen requirements at 36 weeks of postmenstrual age), significant patent ductus arteriosus (requiring surgical or pharmacological closure), severe retinopathy of prematurity (stage 3 or higher retinal vasculopathy), severe brain injury (grade 3 intraventricular hemorrhage, parenchymal hemorrhagic infarction and/or moderate to severe white matter injury) and late-onset sepsis (systemic signs of infection and isolation of a bacterial pathogen in blood culture after 5 days of life). The absence and presence of the disease were assigned a value of 0 and 1, respectively, with a total index range from 0 to 6. Perinatal data were obtained from medical records.

### 2.3 Procedure

Preterm children were recruited for the PRETERM Health and Development Follow-up Project, approved by the Bioethics Committee of the authors’ university. In collaboration with the hospital, families were contacted to inform them about the research. A face-to-face meeting was arranged, where the parents filled and signed the informed consent. The researchers conduct an interview where the demographic data of family and children are collected. Subsequently, in separate rooms, one researcher with the child and one with the parent or parents administered the questionnaires and answered any questions that they had, while they completed the questionnaires on their own. The measure referred to children was completed by agreements between both parents or, in the case of absent father, only by the mother. All other questionnaires were completed individually, although only data collected from the mother are included here.

Regarding attrition, 26.13% of the surviving sample (*n* = 199) did not participate in the study. There were no significant differences concerning sex, GA and BW between participants and non-participants (see Table 2).

**Table 2:** Distribution of sex, gestational age and birth weight in participants and non-participants.

	Participants	Non-participants	*χ^2^*/*t*	*p*	Phi/ Cohen’s *d*
Female	70 (47.62%)	28 (53.85%)	0.596	0.440	0.055
Male	77 (52.38%)	24 (46.15%)			
Gestational age	29.8 (2.2)	30.3 (1.9)	1.510	0.066	0.244
Birth weight	1,288 (362)	1,355 (328)	1.172	0.121	0.189

***p < 0.001; **p < 0.01; *p < 0.05.

### 2.4 Data analysis

IBM SPSS Statistics v29, PROCESS macro (Hayes, 2022) for SPSS, and Jamovi software were used for statistical analyses.

According to the missing-value analysis, 25 (17%) participants had at least one missing value. In particular, KIDSCREEN-27 showed more missing data. We considered for the imputation the loss of 7% of the total items of the questionnaire and 5% of the loss of the item in the sample (Heymans and Twisk, 2022). A multiple imputation was conducted on the dataset. A missing value analysis was performed in IBM SPSS Statistics v29, estimating the loss by randomization with EM and recovering the data by regression for each set of items in each instrument. Little’s MCAR test was used to assess whether missing values were missing due to chance. The assumption of missing at random (MAR) was confirmed for each measurement (*p* > 0.05). Specifically, four imputations were generated for KIDSCREEN-10, KIDSCREEN-27, Duke-UNC and PSS recovering 2, 16, 2, and 6 participants, respectively.

The attrition in the sample was analyzed. Differences between the participant and non-participant samples were studied with a chi-squared test (sex) and independent t-test for two samples (gestational age and birth weight) (Table 2). Descriptive data from demographic and HRQoL variables were presented as mean and *SD* or frequencies. For KIDSCREEN, *t*-values were used and compared with the normed sample (*M* = 50) with a one-sample *t*-test.

Pearson’s correlations were performed between the variables. Multiple mediation analysis, described by Hayes (2022), was applied to investigate simultaneous mediation effects of maternal stress (*M1*) and functional social support (*M2*), between the NMRI (*X*), SFRI (*X*) and HRQoL (*Y*) shown in the hypothetical model (Figure 1). Other similar models considering other variables and all PedsQL subscales were also tested to find the best fit. Multiple mediation analysis is based on regressions, and effect values are unstandardized regression coefficients, measuring indirect effects rather than temporal mediation. The analysis provides a total effect of the model (*c*, including all the effects over *Y*), a direct effect (*c’*, considering only the effect of *X* over Y) and indirect effects (*axb* considering the effects of the mediators). The indirect effect is the product between the effects of *X* over the mediator/s (paths type *a*), and the effects of the mediators on *Y* or partial effects while controlling covariates in the model (paths type *b*). In this case, PROCESS model type 6 (two mediators) (Hayes, 2022) and 10,000 bootstrap simulations were used in the PROCESS macro for SPSS. Bootstrapping is the base for the calculation of the 95% confidence intervals for the indirect effects (lowest and highest results from the random samples). If confidence interval did not include zero, the effect was considered statistically significant.

## 3 Results

A total of 147 VLBW children were followed up until they reached 8 years of age. Table 3 presents descriptive results of KIDSCREEN-27 and KIDSCREEN-10 scores. The means of our sample were higher than the KIDSCREEN normed mean (*M* = 50, *p* < 0.001). Cohen’s *d* ranged from 0.506 to 0.898, indicating medium-to-high effect sizes (see Table 3).

**Table 3:** Mean self-and parent-reported KIDSCREEN scores and mean differences from the normed mean.

KIDSCREEN domains	Mean (*SD*)	Mean difference	*p*	Cohen’s *d*
KIDSCREEN-10 (self-reported)	55.11 (10.10)	5.11	< 0.001	0.506
KIDSCREEN-27 (parent-reported)				
Physical well-being	57.94 (11.08)	7.94	< 0.001	0.717
Psychological well-being	60.87 (12.16)	10.87	< 0.001	0.894
Autonomy and parental relationship	56.94 (13.72)	6.94	< 0.001	0.506
Social support and peers	56.68 (9.72)	6.68	< 0.001	0.688
School environment	58.57 (10.60)	8.57	< 0.001	0.808
Total score	58.20 (9.13)	8.20	< 0.001	0.898

Correlations between the variables in the study are shown in Table 4. First of all, the scores of self-reported KIDSCREEN-10 showed positive correlations with the scores of parent-reported KIDSCREEN-27, being significant only with the psychological well-being and social support and peers subdimensions. Moreover, non-significant correlations were found between KIDSCREEN-10 and the other variables, except the negative correlation with maternal stress. NMRI did not correlate with KIDSCREEN-27 total scores, although it did correlate with its physical well-being subdimension. Total KIDSCREEN-27 score and the score of some of its subdimensions were also negatively correlated with SFRI and maternal stress. Maternal perceived support correlated negatively with KIDSCREEN-27. SFRI correlated significantly and negatively with functional social support.

**Table 4:** Correlations between KIDSCREEN scores, gestational age, birth weight, socio-family risk index, neonatal medical risk index, functional social support and maternal stress.

	KC10	KP27 T	KP27 Ph	KP27 PW	KP27 Pa	KP27 Pe	KP27 Sc	GA	BW	SRI	NMRI	FSS	MS
KC10													
KP27 T	0.126												
KP27 Ph	0.077	0.770**											
KP27 PW	0.187*	0.846**	0.642**										
KP27 Pa	−0.006	0.793**	0.427**	0.567**									
KP27 Pe	0.162*	0.763**	0.517**	0.511**	0.522**								
KP27 Sc	0.106	0.805**	0.505**	0.621**	0.545**	0.567**							
GA	−0.039	−0.001	0.110	−0.062	−0.150	−0.075	0.077						
BW	−0.105	−0.010	0.022	−0.030	−0.149	0.088	0.081	0.688**					
SRI	−0.103	−0.187*	−0.164*	−0.179*	−0.103	−0.112	−0.195*	0.033	−0.078				
NMRI	−0.082	0.153	0.193*	0.025	0.165*	0.064	0.078	−0.120	−0.275**	−0.011			
FSS	0.133	0.157	0.212*	0.183*	−0.035	0.168*	0.136	−0.075	−0.006	−0.195*	0.685		
MS	−0.240**	−0.293**	−0.182*	−0.290**	−0.159	−0.193*	−0.357**	0.056	0.179*	0.044	−0.051	−0.344**	

KC10, KIDSCREEN-10 children; KP27 T, KIDSCREEN-27 parents, total score; KP27 Ph, KIDSCREEN-27 parents, physical well-being; KP27 PW, KIDSCREEN-27 parents, psychological well-being; KP27 Pa, KIDSCREEN-27 parents, autonomy and parent relationship; KP27 Pe, KIDSCREEN-27 parents, social support & peers; KP27 Sc, KIDSCREEN-27 parents, school environment; GA, gestational age; BW, birth weight; SRI, socio-family risk index; NMRI, neonatal medical risk index; FSS, functional social support; MS, maternal stress. **p < 0.01; *p < 0.05.

**Figure 3:**
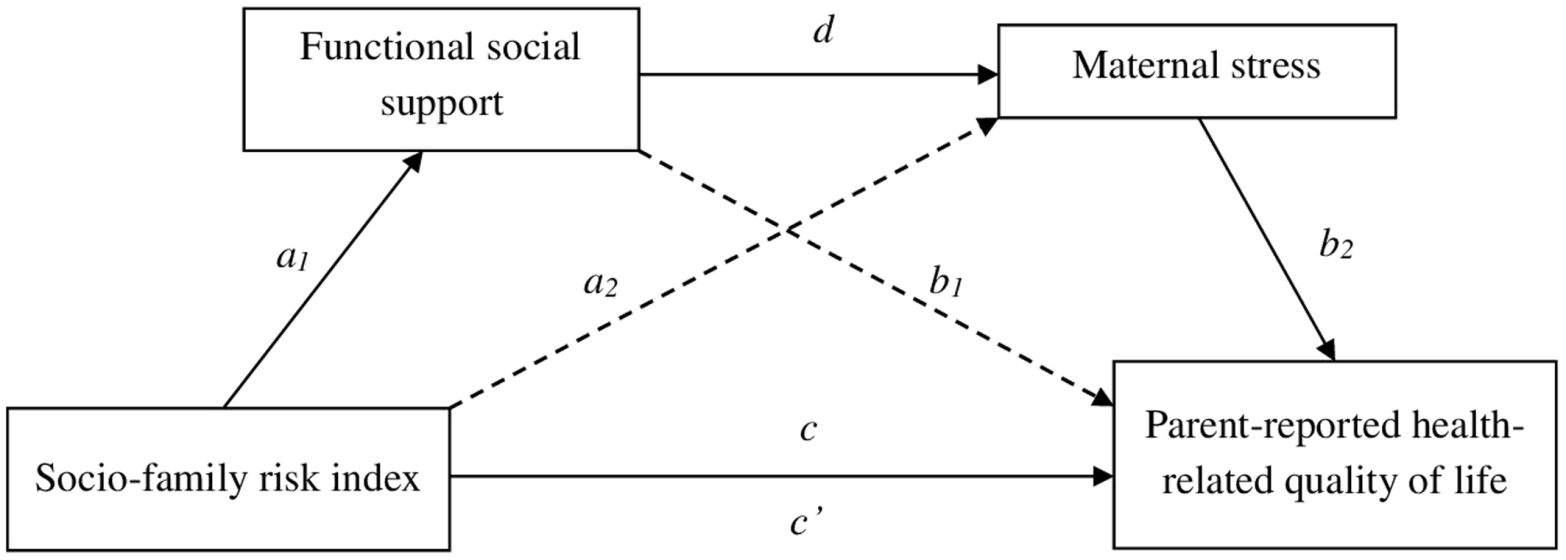
Mediating effect of functional social support and maternal stress in the relationship between socio-family risk and parent-reported health-related quality of life.

Analyses of the initial theoretical model (Figure 1) did not show the expected results. Some relationships between variables were not significant, as was the total effect of the model (*F* = 1.69, *p* > 0.05, *R^2^* = 0.12). Based on these results, two partial models were tested with the two risk indexes. The first partial model (with the neonatal medical risk index) did not show significant direct neither indirect effects between most variables (NMRI with social support, *p* = 0.70; NMRI with maternal stress, *p* = 0.61, NMRI with HRQoL, *p* = 0.08 and social support with HRQoL, *p* = 0.75). The second partial model (with the socio-family risk index) was validated, and it is graphically presented in Figure 3.

Table 5 and Figure 3 show the results of the mediation analysis with the direct and indirect effects. It is shown in Figure 3 that there was a significant influence of SFRI and functional social support (*a_1_* = −0.71, *p* = 0.018). Maternal stress scores were explained by functional social support (*d* = −0.31, *p* < 0.001), but not by SFRI (*a_2_* = −0.08, *p* = 0.762). Furthermore, SFRI (*c’* = −0.80, *p* = 0.036) and maternal stress (*b_2_* = −0.39, *p* = 0.001) exerted a statistically significant influence on the dependent variable of this model (parent-reported HRQoL). However, this result was not significant for functional social support (*b_1_* = 0.04, *p* = 0.733). Finally, regarding the total model effect, there was a significant influence of SFRI on HRQoL (*c* = −0.88, *p* = 0.023).

**Table 5:** Mediation effects of socio-family risk index on parent-reported health-related quality of life through functional social support and maternal stress.

	β	*SE*	Bootstrap 95% CI	*p*
Socio-family risk index → functional social support (*a_1_*)	−0.71	0.30	−1.30, −0.12	0.018
Socio-family risk index → maternal stress (*a_2_*)	−0.08	0.26	−0.60, 0.44	0.761
Functional social support → HRQoL parents report (*b_1_*)	0.04	0.11	−0.18, 0.25	0.733
Maternal stress → HRQoL parents report (*b_2_*)	−0.39	0.12	−0.63, −0.16	0.001
Total effect of socio-family risk index on HRQoL (*c*)	−0.80	0.38	−1.55, −0.05	0.036
Direct effect (*c’*)	−0.88	0.38	−1.64, −0.12	0.023
Functional social support → maternal stress (*d*)	−0.31	0.07	−0.45, −0.17	< 0.001
Total indirect effect of socio-family risk index on HRQoL	−0.08	0.13	−0.35, 0.18	
Indirect effect 1: socio-family risk index → functional social support → HRQoL parents report	−0.03	0.10	−0.23, 0.18	
Indirect effect 2: socio-family risk index → maternal stress → HRQoL parent-reported	0.03	0.12	−0.21, 0.26	
Indirect effect 3: socio-family risk index → functional social support → maternal stress → HRQoL parent-reported	−0.09	0.14	−0.21, −0.01	

This was carried out using Model 6 (two mediators) and 10,000 bootstrap simulations. HRQoL, health-related quality of life; SE, standard error; CI, confidence interval.

Three possible indirect effects were established in this model (Table 5). The first indirect effect did not explain the relationship between SFRI and HRQoL through social support. The results of the second indirect effect were also non-significant, thus SFRI had no effect on HRQoL through maternal stress. Only the third indirect effect was significant, in which SFRI was related to HRQoL through the indirect effects of the two mediating variables: functional social support and maternal stress.

## 4 Discussion

This study aimed to examine the HRQoL of children born preterm with VLBW at 8 years of age and to explore the mediation effects of maternal variables on the relationship between NMRI, SFRI, and HRQoL. This study adds evidence to the existing results in the preterm child population and illustrates relationships between variables that affect children born preterm and their families.

Contrary to our first hypothesis, we found that self-and parent-reported HRQoL of school-aged VLBW children was better than that of the population norm of KIDSCREEN (Ravens-Sieberer et al., 2007). These findings are in disagreement with those of previous studies; however, few works have examined HRQoL in school-aged VLBW children (Peart et al., 2021; Stahlmann et al., 2009; Vederhus et al., 2010). Huhtala et al. (2016) found that healthy VLBW children at 8 years of age did not differ significantly in HRQoL from a control group. Moreover, Hack et al. (2011) found that self-reported health results ELBW children were comparable to those of normal birth weight children. To explain this result, possible hypotheses to be tested in the future would be the existence of greater family involvement from the beginning due to the condition of their children or the fact that they were attending the early care service (greater professional intervention from the beginning). In fact, a large proportion of the participants in this study received care in early intervention services (physiotherapy, psychology and speech therapy), with two thirds of the sample being discharged between the ages of 0 and 3 years old (Lacalle et al., 2023). It has been found that the higher maternal sensitivity is explained by being in early childhood care (Sánchez-Sandoval and Jiménez-Luque, 2023).

The second hypothesis, related to the negative association of NMRI and SFRI with HRQoL, was only partially confirmed. The correlations between variables indicated that they were related to each other before proposing the predictive models. Regarding children’s self-perception, this was barely associated with SFRI and NMRI, but association was significant in the case of parents’ perception. Thus, the predictive models were carried out on parent-reported HRQoL. Although the use of the SFRI and NMRI is limited in previous literature, some studies yield results that align with those obtained in this work. For example, the systematic review by Jardine et al. (2014) shows that the self-perceptions of children with various medical conditions (including some considered in the construction of the NMRI) are similar to those of their reference group. In contrast, from the families’ perspective, the health-related quality of life of these children tends to be rated lower (Cremeens et al., 2006; Jardine et al., 2014). According to these authors, those differences may be related to many variables, including family-related factors.

With regard to perinatal variables, in contrast to other studies (Vederhus et al., 2010), our preliminary correlation analysis showed that GA did not correlate with HRQoL outcomes. Birth weight did not correlate either, but inconclusive results were found in Vieira and Linhares’ (2016) systematic review, where some studies found correlations and others did not. Finally, the cumulative NMRI did not correlate with the total HRQoL score, only weakly with the subdimension of physical well-being at school age. This is consistent with results found in another study, where VLBW children’s neonatal morbidity was mostly unrelated to HRQoL (Vederhus et al., 2010).

On the other hand, this study confirmed that higher socio-family risk correlates with lower HRQoL, both in their total score and in the physical, psychological, and school well-being subdimensions. Other studies have also shown that different variables included in our SFRI (parental education status, higher socioeconomic deprivation) were negatively associated with children’s HRQoL (Berbis et al., 2012; Chien et al., 2006; Liu, Harding, and for the PIANO Study Team, 2021). The final model demonstrated a direct and negative effect of SFRI on HRQoL. The impact of negative social circumstances on children’s quality of life has also been highlighted in studies of children born at term (Kim et al., 2018; Von Rueden et al., 2006).

The third hypothesis stated that two maternal variables, i.e., maternal stress and perceived social support, could influence the relationship between these risk factors and HRQoL. Maternal stress and social support, which are considered risk and protective factors, respectively, did correlate with different dimensions of children’s HRQoL. Treyvaud (2014) emphasized the importance of social and parental factors in influencing child development. The focus on parental role stress was based on research suggesting a negative impact of high parenting stress on VLBW child outcomes (Huhtala et al., 2012). Although the initially proposed model could not be fully validated, a partial model could be confirmed.

The results showed evidence of the mediating effects of functional social support and maternal stress. A higher SFRI was related to lower scores in functional social support, low perceived social support was associated with higher levels of maternal stress, and finally, high levels of maternal stress involve lower perception of HRQoL in their children. Parents’ social support did not find a significant direct association with HRQoL, as was the case of SFRI with parental stress. The effect of social support on HRQoL is mediated by maternal stress. In a study by Howe et al. (2014), mothers reported high levels of stress, as well as high levels of social isolation and little support from their partners.

It should be noted that the variables included in this study did not correlate with the variables most closely related to preterm births (gestational age and birth weight), and that no statistically significant partial model was found for NMRI. This suggests that, the older the children born preterm, the lower the impact of NMRI on quality of life, and the more important SFRI becomes. These mediating effects need to be studied further, in larger populations and at different ages, to confirm this new hypothesis and establish it with certainty. Vieira and Linhares (2016) suggested in their systematic review that there was a need to identify predictive models that assess mediating and moderating effects on HRQoL outcomes with more challenging statistical analyses in people born preterm. Kim et al. (2023) confirmed evidence of neonatal morbidity effects, although they stated that mediation analyses are limited and only considered two types of morbidities. To the best of our knowledge, there are studies investigating risk and protective factors in children born preterm and their relationship with children’s developmental outcomes, but not the mediating effects of family variables on HRQoL outcomes.

This study provides evidence of how some of these family variables mediate the HRQoL of VLBW children. Even though the children in our sample reported, on average, good HRQoL, the results may be important for targeting interventions to improve the quality of life of preterm children scoring below average. It is difficult to intervene in variables intrinsic to the individual (gestational age, birth weight, medical variables, or socio-family factors) or in the quality of life directly. On the other hand, achieving good practice in intervention on mediating variables that positively or negatively affect children’s HRQoL, such as parental stress (Martínez-Shaw and Sánchez-Sandoval, 2023) or social support (Guralnick, 2012), would significantly improve the lives of these children and their family context.

One of the strengths of this study include both self-and parent-reports of HRQoL. Systematic reviews by Eiser and Morse (2001) and Zwicker and Harris (2008) highlight the importance of obtaining information on HRQoL from various sources whenever possible. This work follows these recommendations, having assessed the HRQoL from both perspectives (children and parents). Another strength is the opportunity to shed light on poorly studied variables in the VLBW population and their relationship with family variables. Risk and protective factors for the adequate development of these children have been studied, but not as much as the influence or weight of certain variables on HRQoL. Greater knowledge about medical, psychological, and family variables and their relationship and influence on quality of life will allow developing better intervention programs by combining efforts on what is important.

The limitations include the small sample size, which may have reduced our statistical power. However, it is close to the recommendations made by (Sim et al., 2022) (*n* = 160). The neonatal medical risk index is based on a composite score and does not take into account the impact of each chronic condition on HRQoL, and these results should be interpreted with caution. The effect of bronchopulmonary dysplasia (Kim et al., 2023) is being studied, but would need to be studied with each of the medical conditions of prematurity. The measures used in this study were self-reported. The use of these type of measures could introduce different sources of bias in the results, such as social desirability, although these instruments have been widely used in research. One reason for the loss of some items could be the size and duration of the global questionnaire for parents. Although the children in this sample were, on average, 8 years old and sufficiently able to express their thoughts and emotions (Kim et al., 2023), some may have found it difficult to understand some items due to their conditions. This is the reason why different versions of the KIDSCREEN were chosen to minimize participant fatigue. This made comparisons between them statistically unfeasible. This study used a prospective two-wave design. Longitudinal designs with only two data collection points may overlook certain temporal changes, whereas multi-wave longitudinal studies with at least three data points provide a more robust approach to capturing changes over time. With only two data points, this mediation analysis cannot confirm temporal order or account for potential confounding variables that may influence outcomes between these two time points, reducing its robustness for a full temporal mediation. Data collection was conducted during the COVID-19 pandemic, which made it difficult to access participants and some families refused to participate. Demographic characteristics of non-participating families are not available, so comparisons with participating families could not be studied. Furthermore, loss to follow-up at 8 years may limit the generalizability of the results, although the distribution of sex, gestational age and birth weight did not differ between participants and non-participants. Moreover, it would be interesting to include participants from other hospitals in Spain and even make comparisons with preterm children and their families from other countries.

It would have been valuable to have a control group of full-term children. Our results may be due to the bias introduced by not having said control group. It should be considered that test norms may be outdated (Wolke and Sohne, 1997), thus the quality of life of these preterm children may be overestimated if there is no contemporary comparison group. This would increase the risk of misidentifying fewer children and families in need of support. Therefore, the argument that a control group in the same geographical area would be needed is reinforced for a future line of research.

This study provides evidence on the HRQoL of VLBW preterm school-aged children, which has been understudied in the literature. Although prematurity is often presented as a major risk factor for child development, this study shows a favorable picture for these children during middle childhood. On average, these children and their parents reported better HRQoL than the mean reference value of the KIDSCREEN scales. Probably, medical advances, as well as the involvement of parents and professionals during the first years of life of these children, have been fundamental in overcoming some initial difficulties. In fact, the possible variability in HRQoL is not explained by variables related to extreme prematurity (gestational age, birth weight) or increased neonatal medical risk. Other circumstances common to other families come into play, such as socio-familial factors and the role of the mothers. Our analyses corroborate that when socio-familial circumstances are more adverse, quality of life in childhood suffers. In addition to this direct effect, the socio-familial circumstances of risk weaken the social support that mothers perceive, thereby escalating the stress with which they face their motherhood, also negatively affecting the quality of life of their children in this way. These findings underline the need to implement social policies that support parenthood, especially in adverse social circumstances; and in families with children with higher medical needs where maternal stress has been found to be higher.

## Data Availability

The raw data supporting the conclusions of this article will be made available by the authors, without undue reservation.
