# Corrigendum: Health-related quality of life in children born preterm at school age: the mediating role of social support and maternal stress

**DOI:** 10.3389/fpsyg.2024.1540278

**Published:** 2025-01-07

**Authors:** Melissa Liher Martínez-Shaw, Kari Anne I. Evensen, Sandra Melero, Yolanda Sánchez-Sandoval

**Affiliations:** ^1^Department of Psychology, Faculty of Education Sciences, University of Cadiz, Cádiz, Spain; ^2^Instituto de Investigación e Innovación Biomédica de Cádiz (INiBICA), Cádiz, Spain; ^3^Department of Clinical and Molecular Medicine, Norwegian University of Science and Technology, Trondheim, Norway; ^4^Children's Clinic, St. Olavs Hospital, Trondheim University Hospital, Trondheim, Norway; ^5^Department of Rehabilitation Science and Health Technology, Oslo Metropolitan University, Oslo, Norway

**Keywords:** preterm children, health-related quality of life, socio-family risk index, social support, maternal stress, mediation analysis

In the published article, there was an error. It concerns the wording of the interpretation of a result in the correlations.

A correction has been made to **Discussion**, paragraph 4. This sentence previously stated:

“Finally, the cumulative NMRI did not correlate with the total HRQoL score, but with the subdimension of physical well-being at school age. The higher the neonatal medical risk, the poorer the physical quality of life during school years. This is consistent with results found in another study, where VLBW children with one or more morbidities had significantly lower HRQoL scores (Huhtala et al., 2016).”

The corrected sentence appears below:

“Finally, the cumulative NMRI did not correlate with the total HRQoL score, only weakly with the subdimension of physical well-being at school age. This is consistent with results found in another study, where VLBW children's neonatal morbidity was mostly unrelated to HRQoL (Vederhus et al., 2010).”

The authors apologize for this error and state that this does not change the scientific conclusions of the article in any way. The original article has been updated.

