# Supplementary material for: Health-related quality of life in children born preterm at school age: the mediating role of social support and maternal stress
Source: Front Psychol. 2024 Dec 2;15:1463804. doi: 10.3389/fpsyg.2024.1463804 (PMC11646732; doi:10.3389/fpsyg.2024.1463804)
Supplement: Supplementary file 1 [file Table_1.docx]

Supplementary File

**Supplemental Table 1.**

*Means and standard deviations of social support and maternal stress scales and its subscales.*

| **Scales and subscales** | **Mean** | **SD** |
| --- | --- | --- |
| Global Social Support^1^ | 46.39 | 7.10 |
| Affective Support^1^ | 20.22 | 3.81 |
| Confidant Support^1^ | 26.16 | 3.78 |
| Global Maternal Stress^2^ | 20.53 | 6.38 |
| Baby’s Rewards^2^ | 5.99 | 1.77 |
| Stressors^2^ | 14.54 | 5.69 |

^1^Higher scores indicate more perceived support. In the Spanish validation, a cut-off point at the 15^th^ percentile for the total score, corresponding to a score ≤ 32, was chosen to divide participants into normal or low support.

^2^Higher score indicates a higher level of parental stress.
